# Capsinoids treatment reduces steatosis with consequent attenuation in the progression of metabolic dysfunction-associated steatotic liver disease in obese rats

**DOI:** 10.1590/1414-431X2026e15127

**Published:** 2026-07-10

**Authors:** L.M. Simmer, F.M. Nunes, K.C.C. Santos, D.A.G. Saiz, K.O. Miranda, E.R. Cordeiro, J.N. Boeloni, D.S. Bocalini, A.S. Leopoldo, A.P. Lima-Leopoldo

**Affiliations:** 1 Programa de Pós-Graduação em Nutrição e Saúde, Centro de Ciências da Saúde, Universidade Federal do Espírito Santo, Vitória, ES, Brasil; 2 Programa de Pós-Graduação em Ciências Fisiológicas, Centro de Ciências da Saúde, Universidade Federal do Espírito Santo, Vitória, ES, Brasil; 3 Departamento de Medicina Veterinária, Centro de Ciências Agrícolas e Engenharia, Universidade Federal do Espírito Santo, Alegre, ES, Brasil; 4 Programa de Pós-Graduação em Educação Física, Centro de Educação Física e Esportes, Universidade Federal do Espírito Santo, Vitória, ES, Brasil

**Keywords:** Liver disease, Obesity, Capsinoids, Steatosis, Metabolic dysfunction-associated steatotic liver disease

## Abstract

Among the morbidities triggered by obesity, metabolic dysfunction-associated steatotic liver disease (MASLD) stands out, with its progression resulting from the deposition of lipid molecules in the liver. There is a growing interest in natural/functional foods as an alternative for improving health, as well as for the treatment and prevention of diseases. Capsinoids (Cap) - bioactive compounds present in peppers of the genus *Capsicum* annuum - have been studied for promoting loss of adiposity and increased caloric expenditure. Therefore, this study aimed to investigate the effects of Cap on liver parameters in obese (Ob) rats induced by a high-fat diet (HFD). Male Wistar rats were initially randomized into two groups: standard diet (SD) and HFD. The protocol lasted 27 weeks, including 19 weeks of induction and maintenance of obesity and 8 weeks of Cap treatment. At week 19, the HFD rats were redistributed into the Obese (Ob) and Obese capsinoids (ObCap) groups, and the ObCap group was supplemented daily with chronic Cap treatment by orogastric gavage (10 mg Cap/kg daily). Cap treatment was not effective in improving adiposity and inflammatory parameters of obesity, although it reduced ghrelin, cholesterol, and hepatic fat accumulation, and attenuated MASLD progression, which may be promising for combating chronic diseases related to lipid metabolism.

## Introduction

According to data from the 2024 World Atlas of Obesity (1), around 3.3 billion adults could be affected by excess weight by 2035, representing an elevation from 42% in 2020 to 54% in 2035. Obesity is defined as the excessive accumulation of fat that can harm health and is characterized as a chronic inflammatory metabolic disease, with potential risk for other morbidities, including type 2 diabetes mellitus, liver disease, certain types of cancer, and even mortality (1).

Changes in dietary patterns, including high consumption of ultra-processed foods that contain high concentrations of sugars and fats (2), are related to increased blood glucose levels, which together with obesity, cause health complications. In addition, studies have shown that physical inactivity, combined with inadequate eating habits, contributes to the rapid progression of chronic non-communicable diseases (NCDs) and their related morbidities (3). Researchers point out that, with the obesity pandemic, there has been a concomitant increase in the prevalence and incidence of liver damage resulting from this clinical condition (4).

Among the comorbidities resulting from excess adiposity is non-alcoholic fatty liver disease (NAFLD). Recently, the American and European Societies for the Study of Liver Diseases announced a new nomenclature: steatotic liver disease associated with metabolic dysfunction (MASLD) (5). This disease is related to hepatic steatosis, characterized by fatty infiltrates in more than 5% of hepatocytes (6). It results from increased lipid supply, *de novo* lipogenesis, and reduced fatty acid oxidation. Its characterization is based on morphophysiological alterations of the liver, encompassing structural damage and impairment of hepatic function (7).

The search for alternatives to improve health conditions is increasing, especially for natural and functional foods that help prevent and/or treat NCDs (8). Among these foods, chili peppers, especially species of the *Capsicum* genus, deserve attention, as they contain a wide variety of bioactive compounds and have been used for various purposes, from condiments to medicines. In therapeutic treatments, it is used to relieve pain, regulate body temperature, as an antioxidant and antimicrobial agent, and even to combat obesity. Studies have highlighted its use as a possible strategy for the prevention and treatment of obesity through the metabolic activation of adipose tissue, resulting in a positive effect on health (9).

Capsinoids (Cap), present in chili peppers of the genus *Capsicum* annuum, are analogous to capsaicin, capsiate, dihydrocapsiate, and nordihydrocapsiate and originate from the same biosynthetic pathway, but are considered non-pungent substances (10). The literature has reported that Cap, similarly to capsaicin, binds to the transient receptor potential vanilloid type 1 (TRPV1) and may have similar effects, such as increasing energy expenditure. Cap has been associated with increased thermogenesis in adipose tissue (11). In addition, red pepper seed extract has been shown to improve triglyceride levels, serum inflammatory markers, and insulin sensitivity, as demonstrated by the glucose tolerance test in diabetic rats (12). However, few studies have evaluated the effects of capsinoids on the liver of animals fed high-fat diets (13).

Snitker et al. (12) showed loss of abdominal fat after treatment with 6 mg of capsinoids for 12 weeks. Another study using a single oral intake of capsinoids (9 mg) reported increased energy expenditure and decreased body fat (13). These changes in lipid metabolism after treatment with capsinoids can be explained by the increased expression of genes involved in lipid metabolism, which can have an effect on cholesterol synthesis. Furthermore, the literature indicates that biomarkers of lipid metabolism in the liver are significantly stimulated after the ingestion of capsinoids (14).

Given the evidence that capsinoids may affect obesity and its consequences, specifically in the liver, and the lack of studies on MASLD, the aim of the present study was to investigate whether capsinoid treatment provides benefits in inflammatory and hepatic parameters in obesity. The hypothesis is that chronic administration of capsinoids positively modulates cholesterol, attenuating the progression of MASLD in rats subjected to obesity.

## Material and Methods

### Animal care

Male Wistar rats (30 days old, ≅100 g) were obtained from the Central Animal House of the Federal University of Espírito Santo (Brazil). The animals were housed in individual cages with a controlled environment in terms of light (12 h of inverted light/dark cycle rhythm starting at 9 am), clean-air room temperature (24±2°C), and relative humidity (55±5%). All experiments and procedures were carried out in accordance with the Guide for the Care and Use of Laboratory Animals published by the U.S. National Institutes of Health and current Brazilian legislation. The experimental protocol was approved by the Ethics Committee of the Federal University of Espírito Santo (number: 08-2022).

According to the literature, the characterization of MASLD is based on the different pathophysiological stages of the liver condition, in addition to other conditions such as obesity, insulin resistance (IR), and impaired glucose tolerance (15). These observations were considered in the current study and corroborated other studies, which attribute the prevalence of MASLD to lifestyle changes, primarily overnutrition from a high-fat diet (HFD) and IR, which promote increased lipolysis. This leads to the breakdown of triglyceride (TG) stores and results in increased release of glycerol and free fatty acids (FFAs) into the circulation, which ultimately accumulate in hepatocytes, resulting in the development of MASLD (16). Limitations of this study included the low dose of capsinoids used, even with chronic administration, the vehicle (water by orogastric gavage), the administration time, and the type of compound used, which was not the pure, isolated substance. Furthermore, our biochemical analyses were restricted to plasma and were not measured directly in liver tissue.

### Experimental design

Rats were submitted to a 7-day acclimatization period and then randomized into two groups: standard diet (SD, n=50) and saturated high-fat diet (HFD, n=51). The SD group was fed a standard diet (AIN-93) containing 9.4% lipids (100% soybean oil), 75.7% carbohydrates, and 14.9% proteins, composed of corn starch, casein, dextrinized starch, sucrose, soybean oil, microcrystalline cellulose, mineral mix, vitamin mix, L-cystine, BHT, and choline bitartrate (Pragsoluções Biociências^®^, Brazil). HFD animals were fed a high-fat diet that also followed the same recommendations, but with the addition of lard and was composed of 45.3% lipids (11.5% soybean oil and 88.5% lard), 40.3% carbohydrates, and 14.4% proteins (Pragsoluções Biociências^®^). The caloric density of the diets was 3.81 (kcal/g) for the SD and 4.82 (kcal/g) for the HFD.

All animals had free access to water, and 40 g of pelleted feed was offered per day; the amount not consumed was measured after 24 h for subsequent calculation of caloric intake (daily food consumption = 40 g minus the amount of food not consumed). Caloric intake was calculated by multiplying daily food consumption by the caloric value of each diet (g × kcal). Feed efficiency was calculated by dividing the total weight gain of the animals (g) by the total ingested energy (kcal).

**Figure 1:**
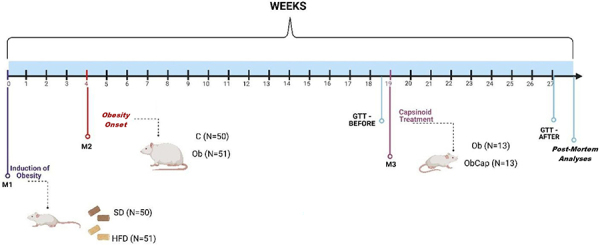
Schematic representation of the experimental design (27 weeks). Experiment 1: SD: standard diet (n=50); HFD: high-fat diet (n=51); Experiment 2: Ob: obese (n=13); ObCap: obese with capsinoids (n=13); GTT: glucose tolerance test; M1: induction of obesity for characterization of obesity onset (4 weeks); M2: maintenance of obesity (week 4 until week 19); M3: treatment with capsinoids for 8 weeks.

During the experimental protocol, body weight (BW) was recorded weekly. The protocol consisted of 2 experiments lasting 27 weeks: *Experiment 1* involved obesity induction (until week 4) and maintenance (week 4 to week 19), and *Experiment 2* began after week 19th, when the groups were redistributed, and the Cap treatment was administered for 8 weeks (Figure 1).

### Experiment 1

#### Obesity induction and maintenance

Obesity was determined when the BW of the HFD group showed a significant increase compared to the SD group. After obesity onset (week 4), the SD and HFD groups were renamed as control (C) and obese (Ob) groups, respectively (Figure 1). Ob rats were maintained in obesity for 15 weeks, then randomized into two different groups: obese (Ob=13) and obese with capsinoids (ObCap=13) (Figure 1). Both groups continued to receive a HFD until the end of the experimental protocol (27 weeks). Considering that this study aimed only to evaluate the effects of capsinoid treatment on obesity, the C rats were excluded from experiment 2.

### Experiment 2

#### Treatment with capsinoids

The ObCap group was supplemented daily with capsinoids (Infinity Pharma, Brazil) (capsiate, dihydrocapsiate, and nordihydrocapsiate) by orogastric gavage (10 mg of capsinoids/kg of BW diluted in 1 mL of water/kg of BW) for 8 weeks. The capsinoid dose was adjusted weekly according to the change in BW to maintain a constant dose throughout the study. The Ob group received the same volume of vehicle by gavage.

### Euthanasia

At the end of the experimental protocol (27 weeks), following a 6-h fasting period, the animals were heparinized and anesthetized with a solution containing ketamine hydrochloride (90 mg/kg) and xylazine hydrochloride (10 mg/kg). Animals that still exhibited signs of nociceptive reflex after anesthetic induction, an anesthetic overdose (lethal dose) was administered, consisting of three times the initial doses of ketamine hydrochloride and xylazine hydrochloride. Following euthanasia, the animals were submitted to a median thoracotomy to collect blood and adipose and hepatic tissue samples.

### Characterization of obesity

To assess obesity, BW was checked weekly. Body fat was determined by summing epididymal, retroperitoneal, and visceral fat. Finally, the adiposity index (AI) was calculated with the formula: [AI = amount of body fat / final BW × 100].

### Comorbidities associated with obesity

To assess the possible metabolic and hormonal changes induced by obesity, the glucose tolerance test (GTT), the homeostatic model assessment for insulin resistance (HOMA-IR) index, and the lipid, hormonal, and inflammatory profile were evaluated.

On weeks 19 and 27 of the experimental protocol, the animals underwent a GTT. Rats were fasted for 6 hours to analyze glucose levels at 30, 60, 90, and 120 min under baseline conditions and after glucose overload (50% glucose; *ip*) (17). The area under curve (AUC) for glucose was also assessed to identify glucose tolerance. HOMA-IR index was used to determine IR, calculated using the following formula: [fasting insulin concentration (μU/mL) × fasting glucose (mmol/L) / 22.5].

For the analysis of lipid, hormonal, and inflammatory profiles, blood samples were collected in Falcon tubes and centrifuged at 8944 *g* for 10 min at 4°C and then stored at -80°C. Insulin (Elabscience Biotechnology, USA), leptin (R&D Systems, USA), ghrelin (Elabscience Biotechnology), glucagon (R&D Systems), adiponectin (Elabscience Biotechnology), interleukin-10 (Elabscience Biotechnology), interleukin-6 (Elabscience Biotechnology), and tumor necrosis factor alpha (TNF-α) (R&D Systems) were determined by enzyme-linked immunosorbent assay (ELISA) using specific kits.

Plasma concentrations of triglycerides (TG), total cholesterol (TC), and high-density lipoproteins (HDL) were determined using specific kits (Bioclin^®^, Brazil) and analyzed using the BS-200 automated biochemical apparatus (Mindray, Brazil).

Liver damage was evaluated through plasma hepatic enzymes glutamic-pyruvic transaminase (TGP), glutamic-oxaloacetic transaminase (TGO), gamma glutamyl transferase (GGT), and lactate dehydrogenase (LDH). The analyses were carried out using specific kits (Bioclin^®^) and analyzed using the BS-200 automated biochemical apparatus.

### Morphology of adipose and hepatic tissues

Morphometry of retroperitoneal and visceral adipose tissues was performed. The collected samples were fixed in 4% paraformaldehyde in PBS (pH 7.4), dehydrated in ethanol, clarified in xylene, and embedded in paraffin at 65°C. The embedded tissues were cut on a rotary microtome (Leica^®^ RM 2125 RTS, Germany) and 5-µm sections were obtained, which were stained with hematoxylin and eosin (HE). For the histological analysis of the adipocyte area, 10 fields from each slide were analyzed to quantify the cellular area (hypertrophy) and number (hyperplasia) of adipocytes. Morphometric analysis for each tissue and experimental group was performed blindly. Images were captured using a 10× objective and a video camera (LAS EZ^®^, ICC50 HD - 51112061, Leica) coupled to an optical microscope (Leica^®^, RM 2125 RTS). Measurements were performed using ImageJ Pro-Plus^®^ software (Media Cybernetics, USA). The area was calculated as the mean value of the area in all measured fields for each group.

For hyperplasia analysis, the mean adipocyte volume was calculated using the equation:

$$\left(\pi /6)\times (3\sigma^{2}\times d^{-}+d^{{-}_{3}}\right)$$ (Eq. 1)

where d- is the mean adipocyte diameter and σ is the standard deviation of the diameter (18). The volume is reported in picoliters (pL). Then, the adipocyte density (0.92 g/mL) was used to determine the fat cell mass. The number of adipocytes was calculated by dividing the total tissue mass (in pL) by the mean adipocyte mass.

The amount of fat in the liver was determined using the Oil Red technique. After euthanasia, liver tissue samples were fixed in PBS-formaldehyde at pH 7.4, then sectioned and placed in a container filled with gel composed of Tissuetek OCT (Sakura Finetek, USA). Samples were then frozen and stored at -20°C, before being cut in 10-µm sections, and stained using the standard Oil Red protocol for lipids (19).

To analyze MASLD progression, liver tissue samples were previously fixed in 4% paraformaldehyde in PBS (pH 7), dehydrated in ethanol, clarified in xylene, and embedded in paraffin at 65°C. Five micrometer-sections were obtained on a rotary microtome (Leica RM 2125 RTS), stained with HE, and mounted with DPX (Sigma-Aldrich, USA). Images were captured with a 40× objective by the video camera coupled to the optical microscope. The sections were then analyzed blindly by a pathologist and scored according to the rodent scoring system proposed by Qayyum et al. (6).

### Statistical analysis

Data distribution was assessed using the Kolmogorov-Smirnov normality test. The results are reported as means±SD. All data (SD *vs* HFD and Ob *vs* ObCap) were compared using the Student's *t*-test for independent samples. Two-way ANOVA for independent samples was used for BW and GTT evaluation, complemented with Bonferroni's *post hoc* test. The significance level adopted was 5%. The statistical analyses and graphics were conducted using GraphPad Prism 8.0 software (GraphPad, USA).

## Results

### Experiment 1

**Figure 2:**
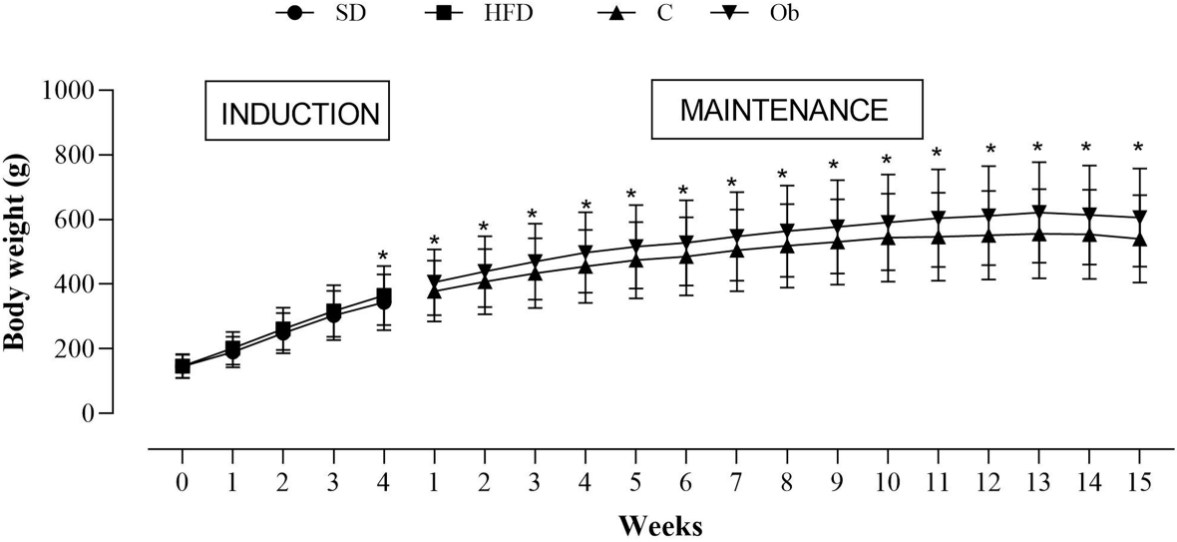
Evolution of body weight during the obesity characterization period. Groups: SD: standard diet (n=50); HFD: high-fat diet (n=51). After the onset and characterization of obesity, the groups were renamed as C: control (n=50) and Ob: obese (n=51). Data are reported as means±SD. *P<0.05, HFD *vs* SD or Ob *vs* C. Two-way ANOVA for independent samples followed by Bonferroni *post hoc* test.

Figure 2 shows the progression of body weight during the period of exposure to experimental diets. From the 4th week onward, the HFD group had significantly greater body weight than the SD group, marking the onset of obesity. The period between the 5th and 19th weeks was designated the obesity maintenance period, and the groups were renamed Control (C) and Obese (Ob), respectively.

At the end of week 19, the final BW (FBW) was higher in the Ob group (606±65 g) than in the C group (541±52 g) (Table 1). In addition, the BW gain was increased in the Ob group, representing a 23.3% elevation. The Ob group also had higher calorie intake, feed efficiency, and AUC for glucose compared to the C group (Table 1), but showed a reduction in food consumption.

**Table 1:** General characteristics of the groups after 19 weeks.

Variables	Control (n=50)	Obese (n=51)
Initial body weight (g)	377±31	405±38*
Final body weight (g)	537±43	604±66*
Food consumption (g/day)	21.22±1.85	17.86±2.26*
Caloric intake (Kcal/day)	80.84±7.04	86.02±10,81*
Feed efficiency (%)	1.89±0.44	2.20±0.37*
AUC	955.71±120.65	1172.58±147.38*

AUC: area under the curve for glucose. Obesity induction lasted 4 weeks and the obesity maintenance period lasted the following 15 weeks. Data are reported as means±SD. *P<0.05, Student's *t*-test.

### Experiment 2

Body weight progression was similar between the Ob and ObCap groups over the 8 weeks (data not shown). ObCap group showed no difference in initial BW (IBW) and FBW, as well as in food consumption, calorie intake, feed efficiency, body fat, and adiposity index, although it did show a greater retroperitoneal fat pad compared to the Ob group (Table 2).

**Table 2:** Nutritional characteristics and adiposity parameters after treatment with capsinoids.

Variables	Obese (n=13)	ObCap (n=13)
Initial body weight (g)	593±51	614±81
Final body weight (g)	583±51	619±70
Food consumption (g/day)	15.10±1.52	15.15±2.28
Caloric intake (Kcal/day)	72.77±7.34	73.03±10.98
Feed efficiency (%)	0.29±0.97	0.06±0.95
Epididymal fat (g)	11.56±1.83	13.08±2.49
Retroperitoneal fat (g)	20.62±4.82	26.08±9.70*
Visceral fat (g)	11.70±2.32	12.76±2.69
Body fat (g)	43.88±7.86	52.42±13.98
Adiposity index (%)	7.52±1.11	8.62±1.83

ObCap: Obese treated with capsinoids. The treatment period lasted 8 weeks. Data are reported as means±SD. *P<0.05, Student's *t*-test for independent samples.

**Figure 3:**
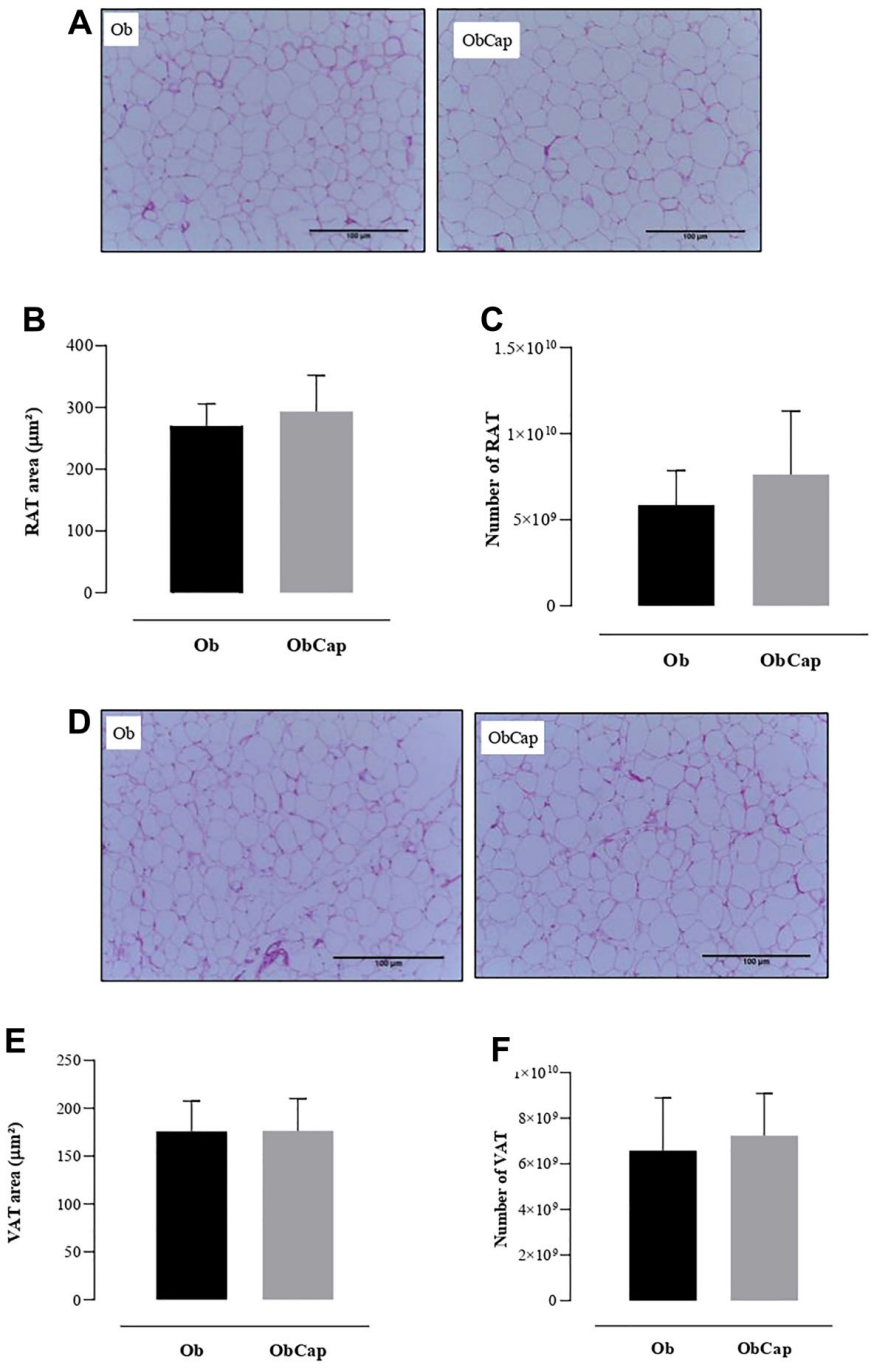
Adipose tissue morphometry. **A**, Retroperitoneal adipose tissue (RAT) representative images in Ob and ObCap groups. **B**, RAT area of Ob and ObCap groups. **C**, Number of RAT cells in Ob and ObCap groups. **D**, Visceral adipose tissue (VAT) representative images in Ob and ObCap groups. **E**, VAT area of Ob and ObCap groups. **F**, Number of VAT cells in Ob and ObCap groups. Data are reported as means±SD. Student's *t*-test. There were no significant differences between groups. Scale bar=100 µm. Ob: obese (n=13); ObCap: obese treated with capsinoids (n=13).

The morphometry of the adipose tissue of the treated groups is shown in Figure 3. There was no treatment effect on the modulation of retroperitoneal (Figure 3B and C) and visceral adipose tissues (Figure 3E and F), since no statistical differences were observed in adipocyte size and number in fat pads.

The ObCap group showed higher glycemic levels at 30 and 90 min (data not shown). However, no significant differences were observed in AUC for glucose and HOMA-IR index, respectively (Table 3). Plasma ghrelin and cholesterol levels were reduced in the ObCap group compared to the Ob group (Table 3). The other parameters were similar between groups.

**Table 3:** Glycemic, inflammatory, hormonal, lipid, and hepatic profiles of the experimental groups.

Variables	Obese (n=13)	ObCap (n=13)
Insulin (pg/mL)	80.81±19.87	73.05±11.32
AUC	1023±105.41	1050±180.42
HOMA-IR	0.57±0.14	0.62±0.09
Leptin (pg/mL)	2897±305.4	3030±375.1
Glucagon (ng/mL)	0.12±0.04	0.12±0.03
Ghrelin (ng/mL)	2.18±1.01	1.04±0.44*
Adiponectin (ng/mL)	43.52±10.13	45.54±5.50
IL-6 (ng/mL)	4.20±0.64	4.45±0.94
IL-10 (ng/mL)	15.30±4.53	20.22±7.04
TNF-α (ng/mL)	31.01±2.92	30.86±2.70
Cholesterol	70.75±6.79	57.50±18.24*
Triglycerides	32.11±17.10	19.50±6.41
HDL	18.17±3.27	17.50±5.33
GPT	88.11±17.62	77.93±18.12
GOT	50.17±14.62	40.50±13.70
GGT	11.45±4.87	13.19±1.13
LDH	285.25±184.08	221.39±83.60

ObCap: Obese treated with capsinoids; AUC: area under the curve for glucose; IL-6: interleukin-6; IL-10: interleukin-10; TNF-α: tumor necrosis factor alpha. HDL: high-density lipoprotein; GPT: glutamic-pyruvic transaminase; GOT: glutamic-oxaloacetic transaminase; GGT: gamma glutamyl transferase; LDH: lactate dehydrogenase. Data are reported as means±SD. *P<0.05, Student's *t*-test for independent samples.

**Figure 4:**
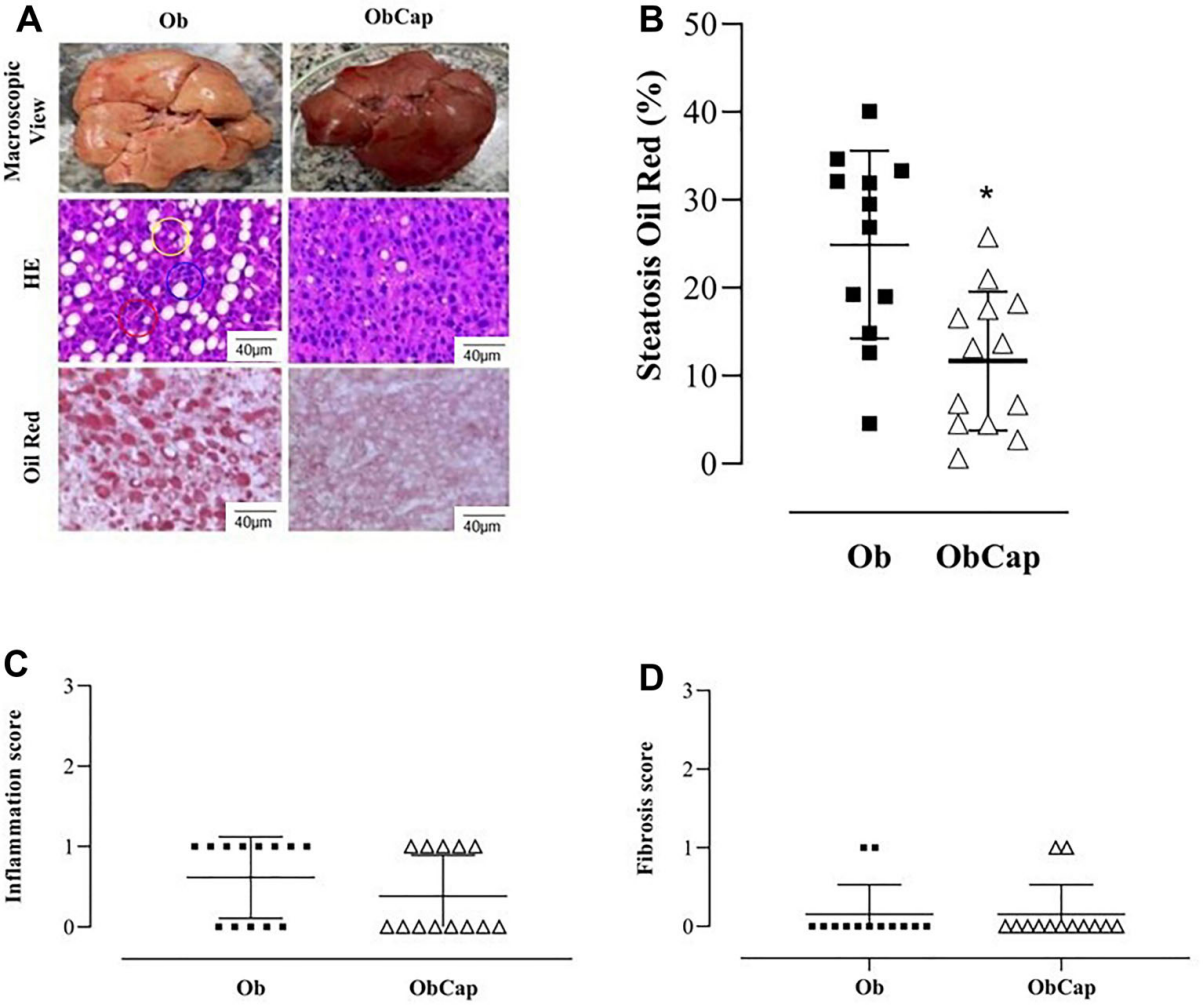
**A**, Representative photographs of the liver, showing macroscopic and microscopic views. The slides were stained with hematoxylin and eosin (HE, 40×, scale bar 10 μm) and Oil Red (40×, scale bar 10 μm). **B**, Assessment of % fat (steatosis) in liver tissue, stained with Oil Red (obese: Ob=12; obese treated with capsinoids: ObCap=13). **C**, Inflammation score of liver tissue indicating clusters of inflammatory cells. **D**, Fibrosis score. **C** and **D**, Ob=13 and ObCap=13. Yellow circle represents HE staining of steatosis; red circle, inflammation; blue circle, fibrosis. Data are reported as means±SD. *P<0.05, Student's *t*-test.

Macroscopic and microscopic aspects of the liver tissue are shown in Figure 4. Animals treated with capsinoids (ObCap) presented a significant reduction in liver fat compared to the Ob group (Figure 4B), which may indicate an attenuation of liver disease progression. Figure 4C and D show the inflammation and fibrosis scores, respectively. There was no difference between the experimental groups.

**Figure 5:**
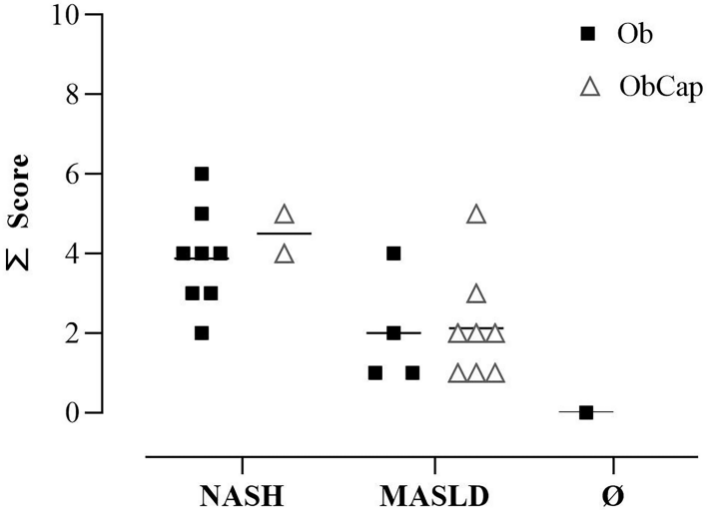
Diagnosis and severity of non-alcoholic steatohepatitis (NASH) and metabolic dysfunction-associated steatotic liver disease (MASLD) in obese (Ob) and obese treated with capsinoids (ObCap) groups according to the diagram proposed for rodents. Ø: Without MASLD.

The results of MASLD diagnostic and severity scores showed differences between groups only in descriptive and percentage terms (Figure 5). Sixty-one percent of the Ob rats (8 out of 13) were diagnosed with non-alcoholic steatohepatitis (NASH), which can be attributed to obesity combined with insulin resistance. This leads to increased hepatic lipogenesis and fat accumulation, exacerbating liver dysfunction. Notably, 61.5% of ObCap rats (8 out of 13) reversed the NASH condition and regressed to MASLD, demonstrating the action of capsinoids on liver tissue and the attenuation of MASLD progression.

## Discussion

The aim of this study was to investigate whether treatment with capsinoids improves inflammatory and liver parameters in obese rats. The main findings were that chronic treatment with capsinoids in obesity did not influence the inflammatory condition. However, treatment with capsinoids attenuated the progression of MASLD, through a lower deposition of lipids in hepatocytes.

Obesity experimental models have been extensively used to study the mechanisms related to NCDs because of the metabolic and physiological similarities between Wistar rats and humans. A diet rich in fat, especially saturated fat, can lead to obesity and induce inflammatory responses in the body (20). Previous research carried out in our laboratory, using an experimental model of HFD-induced obesity, indicates that obesity develops when fat provides more than 30% of total calories (21) and becomes evident after a period typically longer than four weeks of HFD feeding. The current study corroborated these findings by showing a higher body weight in the HFD-fed group from the 4th week onward, indicating the onset of obesity. After obesity onset, a lower food consumption was observed, but with higher caloric intake and feed efficiency, suggesting that the energy consumed was converted more efficiently into body weight. Similar results have been described by other researchers, including previous studies by our research group (21,22).

Morbidities often associated with obesity, such as glucose intolerance, were also observed in the current study, corroborating previous studies. The HFD-fed animals had higher blood glucose levels at all time points, corroborating the literature that shows hyperglycemia in animals fed a diet rich in saturated fat (23). The high blood glucose levels suggest that the obesity model in this study triggered glucose intolerance, which can be attributed to low production of or resistance to insulin (22).

Obesity is often associated with dyslipidemia, characterized by changes in lipid metabolism, especially changes in plasma levels of TG and/or cholesterol (24). However, our results did not show changes in TG and HDL levels, and obesity promoted only hypercholesterolemia (data not shown). The fat used in the HFD was lard, which was probably responsible for the increased cholesterol levels. Research indicates that, with the obesity pandemic, there has been a concomitant increase in the prevalence of liver disease. Research points out that these conditions affect approximately 75% of individuals with obesity and can present risk factors such as excess body fat, insulin resistance, and dyslipidemia, which are considered precursors of liver disease (25).

The Cap treatment did not influence the nutritional profile, which can be partially explained by the hormonal concentrations of leptin and ghrelin found. However, there was a significant reduction in ghrelin levels in the ObCap group, which could result in a greater satiety effect, with a concomitant reduction in food consumption due to the action of capsinoids. Studies have shown an increase in glucagon-like peptide-1 (GLP-1) levels and a tendency to reduce ghrelin levels 15 min after acute administration of capsaicin in the meal, but with no effect on satiety (26). The authors reinforce the need to design studies taking into account issues such as the dose, frequency, duration, as well as the different forms of capsinoid administration in order to provide a better understanding of the effects. Furthermore, another study (27) indicates an improvement in bioenergetic efficiency in rats after administration of single doses of 10 and 100 mg capsiatum in the experimental groups.

Cap has shown positive effects in body fat pads, controlling and reducing obesity (28). However, our findings demonstrated that the Cap treatment had no influence on adiposity parameters, except for the amount of retroperitoneal fat, which was greater in the ObCap group, even though no tissue hypertrophy or hyperplasia was observed. This finding corroborates recent data from our research group (29). A possible explanation lies in the composition of the formulation used. The bioavailability of capsinoids can be significantly affected by factors such as the type of vehicle (lipid or aqueous), the presence of emulsifiers, encapsulation, and chemical stability (30). According to Kim et al. (31), different capsinoid formulations had different effects on visceral adiposity in Wistar rats, suggesting that the delivery method directly interferes with the compound's metabolic action. Furthermore, the predominant type of capsinoids in the formulation can influence the results. Capsiate, for example, has a greater affinity for TRPV1 receptors and greater thermogenic potential, while nordihydrocapsiate has less pungency and reduced efficacy (32). These findings are important because they demonstrate that not all fat pads may respond equally to capsinoid treatment and the need for further research in different adipose tissues.

This result differs from findings from the literature involving human studies, which indicate the effect of capsinoids in reducing body fat pads. Snitker et al. (12), using treatment with 6 mg of capsinoids for 12 weeks, found a reduction in abdominal fat. In addition, Saito et al. (13) suggest that a single oral intake of capsinoids (9 mg) resulted in an increase in energy expenditure and a decrease in body fat. However, the capsinoid supplementation used in the current study was not effective in modulating adipose tissue in rats with HFD-induced obesity. This may be due to the dose and frequency of capsinoids, as well as the effects associated with the different forms of administration.

Treatment with capsinoids was not able to improve glucose intolerance or insulin resistance assessed by HOMA-IR in obesity. These results differed from the study by Kim et al. (11), in which the use of concentrated red pepper seed extract (not specific capsinoids) via gavage in diabetic mice showed improvement in glucose uptake and insulin sensitivity.

A decrease in plasma cholesterol levels was observed in the ObCap group, with no changes in HDL and TG. Using chili pepper extract, Al-Jumayi et al. (33) observed a similar behavior of reduced total cholesterol levels in plasma. Possible explanations could be related to changes in lipid metabolism after treatment with capsinoids through the increased expression of genes involved in lipid metabolism, such as 3-hydroxy-3-methyl-glutaryl-CoA reductase (HMG-CoA reductase), carnitine palmitoyl transferase I (CPT-1), and fatty acid translocase of differentiation 36 (FAT/CD36) (14).

Increased fat deposition was observed in the liver of the Ob group, which was reduced by Cap treatment (Figure 4B). Ohyama et al. (34), investigating C57BL/6J male mice supplemented with dihydrocapsiate, observed significant suppression in hepatic steatosis. Such finding is supported by the work of Baboota et al. (35), which indicates that dihydrocapsiate reduced hepatic triglyceride concentrations, normalized the expression of genes regulating lipid metabolism in the liver, and prevented hepatic steatosis, reinforcing pepper as a potential food ingredient for the management of metabolic changes induced by high fat diets. A possible explanation for the reduction in fat deposition may be related to increased cholesterol synthesis and utilization, resulting in lower circulating levels, or to specific properties of capsinoids that reduce hepatic lipid deposition.

Liver tissue can absorb free fatty acids and synthesize them into triglycerides, which are then stored as fat. Excess fatty acids lead to insulin resistance, worsening MASLD. As in humans, we observed these factors in the current study. The gold standard for diagnosing this condition in humans is biopsy followed by histological analysis, which provides a diagnostic score. Understanding the mechanisms involved is crucial. Our study advances the understanding of lipid metabolism in hepatocytes, offering new insights into hepatic lipid metabolism. Additionally, other studies present genetic factors that can be addressed in future studies, with more in-depth analyses of the mechanisms involved in MASLD progression (36,37).

Liver disease can be classified according to its several stages, starting with hepatic steatosis, represented by the isolated accumulation of fat in the liver, progressing to NASH, defined by the association of steatosis and inflammation, resulting in liver damage, accompanied or not by fibrosis (5). Obesity can lead to inflammation by impairing insulin signaling, which is reflected in the activation of intermediate inflammatory pathways through pro-inflammatory cytokines (38). However, in the current study, Cap treatment did not influence the levels of adiponectin, IL-6, IL-10, and TNF-α. In contrast, Sinisgalli et al. (39) reported an improvement in systemic inflammation after the treatment of obesity with red bell pepper consumption, evidenced by the reduction in the expression of pro-inflammatory cytokines. This finding differs from that of Ohyama et al. (34), who reported an improvement in the inflammatory parameters of mice that received dihydrocapsiate, a component of capsinoids, in the diet for 12 weeks.

The results of this study indicated that obesity induced by HFD promoted the development of steatosis and steatohepatitis in animals. In addition, more than 60% of the animals were classified as having NASH, and the European Society for the Study of the Liver recommends lifestyle modifications for its treatment. Excess energy from the diet, probably associated with insulin resistance, plays a crucial role in the accumulation of fatty acids in hepatocytes, facilitating lipogenesis and inhibiting lipolysis (40). Once obesity was established and MASLD diagnostic scores and degree showed steatosis and steatohepatitis, the animals were treated with capsinoids, but no effect was observed in inflammatory cell clusters and fibrosis (Figure 4C and D).

Another important aspect observed in the current study was the attenuation of NAFLD with Cap treatment, unlike what was observed in untreated Ob animals, which developed NASH. This condition may be due to increased uptake of fatty acids from adipose tissue, hepatic synthesis of fatty acids, increased fat consumption, decreased beta-oxidation, or even decreased very low-density lipoprotein (VLDL) (40). Prolonged inhibition of beta-oxidation causes further harm to health, creating a vicious cycle that makes reversing the condition more difficult and leads to progressive deterioration of liver function (27). NASH, also diagnosed in the animals of this study, is a progressive disease that can develop into liver cirrhosis and hepatocellular carcinoma. In the histological characteristics of NASH, although some degree of fibrosis is often present, it is not required for diagnosis. NASH is characterized by specific liver damage, which involves the presence of steatosis, hepatocyte degeneration, and inflammation (16). We believe that this is the first study to investigate the effects of Cap on obesity-induced MASLD.

In summary, the significant reduction in hepatic fat in the group treated with capsinoids, with consequent attenuation of MASLD, unlike that observed in untreated Ob animals, which progressed to NASH, can be attributed to the reduction of lipid droplets in hepatic tissue, suggesting a positive metabolic effect. However, understanding the mechanisms of action of bioactive compounds present in natural and functional foods must be expanded through in-depth analysis of how these substances can be used as effective dietary strategies to aid in the prevention and/or treatment of chronic metabolic diseases.

### Study limitations

The protein expression of key signaling pathways in hepatic tissue was not assessed, which could provide a more detailed understanding of the molecular mechanisms. Another limitation is that this study was conducted in an animal model, and direct extrapolation of these findings to humans should be approached with caution.

## Data Availability

All data generated or analyzed during this study are included in this published article.

## References

[1] World Health Organization (WHO) (2024). World Obesity Federation. Atlas of Obesity.

[2] Louzada MLC, Martins APB, Canella DS, Baraldi LG, Levy RB, Claro RM (2015). Ultra-processed foods and the nutritional dietary profile in Brazil. Rev Saude Publica.

[3] Asrih M, Jornayvaz FR (2015). Metabolic syndrome and nonalcoholic fatty liver disease: Is insulin resistance the link?. Mol Cell Endocrinol.

[4] Younossi ZM, Koenig AB, Abdelatif D, Fazel Y, Henry L, Wymer M (2016). Global epidemiology of nonalcoholic fatty liver disease-Meta-analytic assessment of prevalence, incidence, and outcomes. Hepatology.

[5] Moreira RO, Valerio CM, Villela-Nogueira CA, Cercato C, Gerchman F, Lottenberg AMP (2023). Brazilian evidence-based guideline for screening, diagnosis, treatment, and follow-up of metabolic dysfunction-associated steatotic liver disease (MASLD) in adult individuals with overweight or obesity: a joint position statement from the Brazilian Society of Endocrinology and Metabolism (SBEM), Brazilian Society of Hepatology (SBH), and Brazilian Association for the Study of Obesity and Metabolic Syndrome (Abeso). Arch Endocrinol Metab.

[6] Qayyum A, Nystrom M, Noworolski SM, Chu P, Mohanty A, Merriman R (2012). MRI steatosis grading: development and initial validation of a color mapping system. AJR Am J Roentgenol.

[7] Brazil (2009). Ministry of Health. Functional Foods.

[8] Zhang LL, Liu DY, Ma LQ, Luo ZD, Cao TB, Zhong J (2007). Activation of transient receptor potential vanilloid type-1 channel prevents adipogenesis and obesity. Circ Res.

[9] Ribeiro CSC, Lopes CA, Carvalho SIC, Henz GP, Reifschneider FJB (2008). Brazilian Agricultural Research Corporation. Capsicum peppers.

[10] Okamatsu-Ogura Y, Tsubota A, Ohyama K, Nogusa Y, Saito M, Kimura K (2015). Capsinoids suppress diet-induced obesity through uncoupling protein 1- dependent mechanism in mice. J Funct Foods.

[11] Kim HK, Jeong J, Kang FY, Go GW (2020). Red pepper (*Capsicum annuum* L.) seed extract improves glycemic control by inhibiting hepatic gluconeogenesis via phosphorylation of foxo1 and ampk in obese diabetic db/db mice. Nutrients.

[12] Snitker S, Fujishima Y, Shen H, Ott S, Sunyer XP, Furuhata Y (2009). Effects of novel capsinoid treatment on fatness and energy metabolism in humans: possible pharmacogenetic implications. Am J Clin Nutr.

[13] Saito M, Yoneshiro T (2013). Capsinoids and related food ingredients activating brown fat thermogenesis and reducing body fat in humans. Curr Opin Lipidol.

[14] Hong Q, Xia C, Xiangying H, Quan Y (2015). Capsinoids suppress fat accumulation via lipid metabolism. Mol Med Rep.

[15] Cui XS, Li HZ, Xie CZ, Gao JM, Chen YY, Zhang HY (2025). Rodent model of metabolic dysfunction‐associated fatty liver disease: a systematic review. J Gastroenterol Hepatol.

[16] Brown GT, Kleiner DE (2016). Histopathology of nonalcoholic fatty liver disease and nonalcoholic steatohepatitis. Metabolism.

[17] Mendes BF, Costa-Pereira LV, Andrade JA, Magalhães COD, Pereira RRS, Esteves EA (2022). Superior cardiometabolic and cellular adaptive responses to multiple versus single daily sessions of high-intensity interval training in Wistar rats. Sci Rep.

[18] Eriksson HD, Andersson DP, Bäckdahl J, Hoffstedt J, Rössner S, Thorell A (2015). Adipose tissue morphology predicts improved insulin sensitivity following moderate or pronounced weight loss. Int J Obes (Lond).

[19] Lírio LM, Forechi L, Zanardo TC, Batista HM, Meira EF, Nogueira BV (2016). Chronic fructose intake accelerates non-alcoholic fatty liver disease in the presence of essential hypertension. J Diabetes Complications.

[20] Bojková B, Winklewski PJ, Wszedybyl-Winklewska M (2020). Dietary fat and cancer - which is good, which is bad, and the body of evidence. Int J Mol Sci.

[21] Matias AM, Estevam WM, Coelho PM, Haese D, Kobi JBBS, Lima-Leopoldo AP (2018). Differential effects of high sugar, high lard or a combination of both on nutritional, hormonal and cardiovascular metabolic profiles of rodents. Nutrients.

[22] Ferron AJT, Jacobsen BB, Sant'Ana PG, de Campos DHS, de Tomasi LC, Luvizotto RAM (2015). Cardiac dysfunction induced by obesity is not related to β- adrenergic system impairment at the receptor-signalling pathway. PLoS One.

[23] Álvarez CMM, Gómez-Crisóstomo NP, De la Cruz-Hernández EN, Zazueta C, Aguilar-Gamas CF, Martínez-Abundis E (2023). Differential disruption on glucose and insulin metabolism in two rat models of diet-induced obesity, based on carbohydrates or lipids. Mol Cell Biochem.

[24] Berberich AJ, Hegele RA (2022). A modern approach to dyslipidemia. Endocr Rev.

[25] Cobbina E, Akhlaghi F (2017). Non-alcoholic fatty liver disease (NAFLD) - pathogenesis, classification, and effect on drug metabolizing enzymes and transporters. Drug Metab Rev.

[26] Smeets AJ, Westerterp-Plantenga MS (2009). The acute effects of a lunch containing capsaicin on energy and substrate utilisation, hormones, and satiety. Eur J Nutr.

[27] Kazuya Y, Tonson A, Pecchi E, Dalmasso C, Vilmen C, Fur YL (2014). A single intake of capsiate improves mechanical performance and bioenergetics efficiency in contracting mouse skeletal muscle. Am J Physiol Endocrinol Metab.

[28] Mosqueda-Solís A, Sánchez J, Reynés B, Palou M, Portillo MP, Palou A (2018). Hesperidin and capsaicin, but not the combination, prevent hepatic steatosis and other metabolic syndrome-related alterations in western diet-fed rats. Sci Rep.

[29] Santos KCC, Domingos LF, Nunes FM, Simmer LM, Cordeiro ER, Filetti FM (2024). Capsinoids increase antioxidative enzyme activity and prevent obesity-induced cardiac injury without positively modulating body fat accumulation and cardiac oxidative biomarkers. Nutrients.

[30] Nigam K, Gabrani R, Dang S (2019). Nano-emulsion from capsaicin: formulation and characterization. Mater Today Proc.

[31] Kim JY, Lee MS, Jung S, Joo H, Kim CT, Kim IH (2014). Anti-obesity efficacy of nanoemulsion oleoresin capsicum in obese rats fed a high-fat diet. Int J Nanomedicine.

[32] Gupta R, Kapoor B, Gulati M, Kumar B, Gupta M, Sing SK (2022). Sweet pepper and its principle constituent capsiate: functional properties and health benefits. Crit Rev Food Sci Nutrit.

[33] Al-Jumayi HAO, Elhendy HA, Darwish AMG (2020). Biological effects of red chili pepper (*Capsicum annuum*) consumption on high fat diet female albino rats. Pak J Biol Sci.

[34] Ohyama K, Suzuki K (2017). Dihydrocapsiate improved age-associated impairments in mice by increasing energy expenditure. Am J Physiol Endocrinol Metab.

[35] Baboota RK, Khare P, Mangal P, Singh DP, Bhutani KK, Kondepudi KK (2018). Dihydrocapsiate supplementation prevented high-fat diet-induced adiposity, hepatic steatosis, glucose intolerance, and gut morphological alterations in mice. Nutr Res.

[36] Xiang M, Tian X, Wang H, Gan P, Zhang Q (2024). Inappropriate diet exacerbates metabolic dysfunction-associated Steatotic liver disease via abdominal obesity. Nutrients.

[37] Papatheodoridi M, De Ledinghen V, Lupsor-Platon M, Bronte F, Bousier J, Elshaarawy O (2024). Agile scores in MASLD and ALD: external validation and their utility in clinical algorithms. J Hepatol.

[38] Smith IG, Shankaran M, Yoshino M, Schweitzer GG, Chondronikola M, Beals JW (2020). Insulin resistance drives hepatic de novo lipogenesis in nonalcoholic fatty liver disease. J Clin Invest.

[39] Sinisgalli C, Vezza T, Diez-Echave P, Ostuni A, Faraone I, Hidalgo-Garcia L (2021). The beneficial effects of red sun‐dried *Capsicum annuum* L. cv senise extract with antioxidant properties in experimental obesity are associated with modulation of the intestinal microbiota. Mol Nutr Food Res.

[40] da Ponte IM, Lima MES, Albuquerque MCF, Veloso AFH, Bachur TPR (2020). Non-alcoholic steatohepatitis: a syndrome in evidence. Braz J Health Rev.

